# Regional microglia are transcriptionally distinct but similarly exacerbate neurodegeneration in a culture model of Parkinson’s disease

**DOI:** 10.1186/s12974-018-1181-x

**Published:** 2018-05-11

**Authors:** Eric Wildon Kostuk, Jingli Cai, Lorraine Iacovitti

**Affiliations:** 1 Department of Neuroscience, Farber Institute for Neurosciences, Thomas Jefferson University, Room 320, Bluemle Life Sciences Building, 233 S. 10th Street, Philadelphia, PA 19107 USA; 2 Department of Neurology, Farber Institute for Neurosciences, Thomas Jefferson University, Room 320, Bluemle Life Sciences Building, 233 S. 10th Street, Philadelphia, PA 19107 USA; 3 Department of Neurosurgery, Farber Institute for Neurosciences, Thomas Jefferson University, Room 320, Bluemle Life Sciences Building, 233 S. 10th Street, Philadelphia, PA 19107 USA

**Keywords:** Microglia, Astrocyte, Neuron, Parkinsons’ disease, Neurodegeneration

## Abstract

**Background:**

Parkinson’s disease (PD) is characterized by selective degeneration of dopaminergic (DA) neurons of the substantia nigra pars compacta (SN) while neighboring ventral tegmental area (VTA) DA neurons are relatively spared. Mechanisms underlying the selective protection of the VTA and susceptibility of the SN are still mostly unknown. Here, we demonstrate the importance of balance between astrocytes and microglia in the susceptibility of SN DA neurons to the PD mimetic toxin 1-methyl-4-phenylpyridinium (MPP^+^).

**Methods:**

Previously established methods were used to isolate astrocytes and microglia from the cortex (CTX), SN, and VTA, as well as embryonic midbrain DA neurons from the SN and VTA. The transcriptional profile of isolated microglia was examined for 21 canonical pro- and anti-inflammatory cytokines by qRT-PCR with and without MPP^+^ exposure. Homo- and heterotypic co-cultures of neurons and astrocytes were established, and the effect of altering the ratio of astrocytes and microglia in vitro on the susceptibility of midbrain DA neurons to the PD mimetic toxin MPP^+^ was investigated.

**Results:**

We found that regionally isolated microglia (SN, VTA, CTX) exhibit basal differences in their cytokine profiles and that activation of these microglia with MPP^+^ results in differential cytokine upregulation. The addition of microglia to cultures of SN neurons and astrocytes was not sufficient to cause neurodegeneration; however, when challenged with MPP^+^, all regionally isolated microglia resulted in exacerbation of MPP^+^ toxicity which was alleviated by inhibition of microglial activation. Furthermore, we demonstrated that isolated VTA, but not SN, astrocytes were able to mediate protection of both SN and VTA DA neurons even in the presence of exacerbatory microglia; however, this protection could be reversed by increasing the numbers of microglia present.

**Conclusion:**

These results suggest that the balance of astrocytes and microglia within the midbrain is a key factor underlying the selective vulnerability of SN DA neurons seen in PD pathogenesis and that VTA astrocytes mediate protection of DA neurons which can be countered by greater numbers of deleterious microglia.

**Electronic supplementary material:**

The online version of this article (10.1186/s12974-018-1181-x) contains supplementary material, which is available to authorized users.

## Background

Parkinson’s disease (PD) is a progressive neurodegenerative disease which is characterized by the selective degeneration of dopaminergic (DA) neurons of the substantia nigra pars compacta (SN), whereas the neighboring DA neurons of the ventral tegmental area (VTA) are relatively spared [1]. Previous studies have examined the DA neurons of these two regions to better understand why only one subset of DA neurons is significantly more vulnerable to PD than the other [2–5]. Recently, greater focus has been placed on extrinsic mechanisms, such as the role of astrocytes, in the disease process suggesting that neuronal vulnerability results from both intrinsic and extrinsic influences. Indeed, our recent work (Kostuk et al. 2018, *in submission*) has demonstrated a vast transcriptional difference between SN and VTA astrocytes such that VTA, but not SN, astrocytes release factors which mediate protection of VTA, SN, and induced pluripotent stem cell (iPSC) DA neurons. In addition to astrocytes, the role of microglia, another important glial cell type, in the pathogenesis of PD has become an area of great interest.

Microglia are considered to be the innate immune cells of the brain [6]. Their role in the clearance of damaged cells, such as neurons, and other foreign contaminants of the brain is well established [7–9]. It is well known that microglia derive from a myeloid lineage [10] and that injury or disease can cause increased microglial activation within the brain [11–14]. Indeed, in PD patients and animal models of PD, greater numbers of microglia [15], as well as enhanced microglial reactivity [16, 17], are observed in the SN. In addition to their role as scavengers in the brain, microglia also release pro- and anti-inflammatory molecules in response to insult or injury [9, 18, 19]. Indeed, the canonical microglial activator lipopolysaccharide (LPS) has been used to stimulate microglial activation and the subsequent cytokine release in order to model the immune response in vivo and in vitro [12, 20–22]. Thus, it has been reported that LPS stimulation of microglia within the SN is sufficient to produce DA neurodegeneration [23], suggesting a role for microglia and their factors in disease progression.

Previous studies have additionally examined the interaction of microglia with PD mimetic toxins. For example, rotenone, a pesticide known to be a DA neurotoxin, was shown to require the presence of microglia in order to mediate toxicity of midbrain DA neurons in culture [24]. This raises the possibility that the interaction of PD mimetic toxins with microglia could be a potential pathway by which neurodegeneration occurs. In addition to the potential role that toxins could have on microglia, leading to an exacerbation of toxicity, the ratio of microglia to neurons within the brain could potentially produce different reactions to inflammatory stimuli. Previous work has shown that the regional distribution of microglia throughout the brain is diverse [25, 26], with the SN being populated by a high ratio of microglia to neurons. Furthermore, recent transcriptional profiling of discrete brain regions (cortex, hippocampus, cerebellum, striatum) has suggested that similar to astrocytes, microglia exhibit regionally distinct transcriptional profiles [27]. However, the potential regionality of midbrain microglia has yet to be investigated.

Therefore, in this study, we sought to determine whether midbrain SN and VTA microglia exhibit regionally specific gene expression profiles and whether these regionally isolated microglia can contribute differentially to the susceptibility of SN DA neurons to MPP^+^-induced toxicity as compared to the VTA. Furthermore, we investigated whether the ratio of microglia to neurons to astrocytes within the midbrain plays a role in the selective vulnerability of the SN to PD mimetic toxins. We found that regionally isolated microglia from three distinct brain regions, the cortex, SN, VTA, exhibit differential basal cytokine profiles. Furthermore, when challenged with the PD mimetic toxin MPP^+^, all regionally isolated microglia respond with distinct cytokine profiles. Interestingly, the addition of microglia alone is not sufficient to induce DA neuron death in cultures of either SN or VTA neurons. However, when challenged with MPP^+^, all regionally isolated microglia similarly exacerbate MPP^+^ toxicity, and this exacerbation of toxicity can be alleviated by inhibiting the activation of microglia via pharmacological inhibition of the TLR4 receptor. Finally, we demonstrated that greater ratios of VTA, but not SN, astrocytes are able to protect both SN and VTA DA neurons from MPP^+^ toxicity and that increasing the microglial ratio counters the protective effect of VTA astrocytes. Together, these results suggest that the susceptibility of SN DA neurons to a PD mimetic toxin results from a regional sensitivity to multiple environmental factors, rather than simply from an intrinsic neuronal vulnerability. A greater appreciation of the role of extrinsic mechanisms, mainly the protective nature of astrocytes and deleterious functions of microglia, in disease pathogenesis is important for the consideration of possible therapeutics in PD.

## Methods

### Animals and IACUC policies

All animals used in this study were maintained in accordance with the Office of Animal Resources at Thomas Jefferson University. The protocols were approved by the Institutional Animal Care and Use Committee (IACUC) at Thomas Jefferson University, protocol #457K/01499.

### Embryonic tissue culture

Timed pregnancies were performed using male and female rats from our colony of animals expressing green fluorescent protein under control of the human tyrosine hydroxylase promotor (TH-GFP) [1]. Embryonic day zero (E0) was determined as the first day a vaginal plug was visible. On E14.5, embryos were harvested and placed in ice cold DPBS (Gibco #14190-144; no CaCl2 or MgCl2). GFP+ pups were visualized under a dissection microscope (Nikon SMZ1500; adjustable × 1–× 11.5 objective) by using a high-pressure mercury lamp with a 495-nm filter attachment. The midbrain was surgically extracted and cleaned of non-GFP+ tissues, and the VTA and SN were carefully separated. Tissues were collected and placed in 2.5 mL of enzymatic dissociation solution containing 5 mg DNAse I (Sigma #10104159001), 5 mg Papain (Sigma #10108014001), and 50 mg l-cysteine (Sigma) for up to 30 min. Solution was gently agitated every 5 min to aid in dissociation. NEP basal media (DMEM/F12, B-27 and N2 Supplements (Gibco), 1 mg/mL BSA, and Penn/Strep) was added to terminate the enzymatic reaction. Tissues were then briefly spun at 1000 rpm for 2 min, and the supernatant media was aspirated. Tissues were re-suspended in culture media (NEP basal media + 5% FBS) for 5 min prior to mechanical dissociation. For mechanical dissociation, a 1-mL pippetor was used with low retention tips and tissues were gently triturated eight to ten times. Cells were then pelleted for 5 min at 1000 rpm, re-suspended in culture media, and counted on an automated hemocytometer (Countess FLII Invitrogen). Cells were plated in a microdrop on 96-well tissue culture plates or 8-well chamber slides pre-coated with poly-d-lysine (0.5 mg/mL, overnight at 37 °C) at a density of 5–7 × 10^4^ cells in approximately 20 μL of culture media. Cells were allowed to attach to the substrate before media was added to final volume of 100–200 μL per well. Cells were allowed to mature for 6 days prior to the addition of isolated microglia (see below) in a 1:1 microglia to neuron ratio and allowed to mature for 24 h prior to MPP^+^ treatment. This ratio was established based on previous studies demonstrating similar numbers of MAC1+ or Iba1+ microglia and TH+ neurons within the SN [16, 28]. Experiments where inhibition of microglia was performed utilized the commercially available TLR4 antagonist 2-acetamidopyranoside (TLR4-IN-C34, Sigma Cat# SML0832). On day 7 in vitro (DIV7), C34 was added to cultures 2 h prior to MPP^+^ addition. Cultures were fixed 24–48 h after MPP^+^ treatment, and the full well was imaged using a Nikon Eclipse TI-e fitted with a Photometrics Coolsnap ES2 camera. Images were analyzed in ImageJ using a custom-designed macro for cell quantification. TH-GFP+ cell counts were compared to appropriate non-treated controls.

### Tissue culture—isolated astrocytes and microglia

Astrocytes and microglia were collected using previously published methods [2–4]. Briefly, postnatal day 1–5 GFP+ pups were anesthetized on ice and quickly decapitated. Brains were removed and placed in ice cold DPBS (Gibco). GFP+ brains were visualized under a dissection microscope (Nikon SMZ1500). The midbrain and cortices were carefully dissected away from other brain regions and non-GFP+ tissues of the midbrain were cut away. The SN and VTA regions were carefully separated and collected in ice cold PBS. Whole cortices were collected and dissected into smaller pieces prior to enzymatic digestion. Tissues were then subjected to enzymatic digestion using a trypsin (0.1%)/DNAse I (0.5 mg/mL) mixture for 5 min at 37 °C. The supernatant enzyme was aspirated carefully, and tissues were washed twice in DPBS. Tissues were then mechanically dissociated in 0.5 mg/mL DNAse I and gently triturated eight to ten times. Cells were then pelleted for 5 min at 1000 rpm, re-suspended in astrocyte media (DMEM/F12 + 10% FBS and Penn/Strep) and plated on tissue culture flasks. Cultures were maintained for 2–4 weeks with media changes every other day. To separate astrocytes (adherent) and microglia (non-adherent) for experimental use, flasks were shaken at 220 RPM for 24 h and the non-adherent cells collected. The cell suspension was passed through a 70-mm cell strainer prior to centrifugation at 1000 rpm for 5 min. The cells were re-suspended in culture media for experimental use. Samples of non-adherent cells were stained for the microglial markers Iba1 (allograft inflammatory factor 1 (Iba1, Wako #019-19741, RRID:AB_2665520) and CD11b (BD Biosciences Cat# 550299, RRID:AB_393594) to confirm homogeneity of the culture.

### Co-culture studies: astrocyte to neuron to microglia

Neuron-astrocyte co-cultures were prepared similarly to previously published methods [5]. Briefly, isolated regional astrocytes were trypsinized, dissociated to a single cell suspension, and quantified. Increasing numbers of astrocytes (60,000–120,000) were plated onto 48-well tissue culture plates. Cells were allowed to adhere and grow for 48 h prior to further experimentation. Subsequently, embryonic neuron preparations were carried out to obtain TH-GFP+ neurons. Neurons were plated onto regional astrocytes in a homo- and heterotypic manner at 2:1 and 4:1 (astrocyte to neuron) ratios. These ratios were established based on previous studies examining the number of astrocytes in the midbrain [28, 29], as well as our previous work demonstrating increased ratios of VTA but not SN astrocytes mediate protection from MPP^+^ toxicity (Kostuk et al. 2018, *in submission*). Twenty-four hours after plating, all cultures were treated with 1 μM AraC to halt astrocytic proliferation. Cultures were then allowed to mature for five more days in vitro prior to microglial addition. Isolation and collection of SN and VTA microglia in sufficient numbers for experimental manipulation would require a vast number of animals to be sacrificed; as such, cortical microglia were used as they were easier to obtain in sufficient numbers. Microglia were added in a 1:1 and 2:1 (microglia to neuron) ratio. Twenty-four hours after microglial addition, MPP^+^ was added at 50 μM for 24–48 h. After MPP^+^ treatment, all cells were fixed and GFP+ cells were quantified using the Nikon Eclipse camera system described above.

### RNA isolation and cDNA synthesis

Total RNA was isolated directly from freshly collected cells in TRIzol (Invitrogen), a modification of the guanidine isothiocyanate-phenol-chloroform extraction method. cDNA was synthesized by using at least 1 μg total RNA in a 20-μl reaction with Superscript IV (Invitrogen) and oligo (dT)12–18 (Invitrogen). One microliter of RNase H (Invitrogen) was added to each reaction tube, and the tubes were incubated for 20 min at 37 °C before proceeding to real-time PCR.

### Real-time PCR analysis

Real-time PCR was carried out on the 7500 Real Time PCR System using SYBR green PCR master mix (both from Applied Biosystems). GAPDH was used as an internal control. PCR analyses were conducted in triplicate for each sample. The reaction mix consisted of 6.35 ng cDNA, 0.5 μM forward and reverse primer mix, and × 1 SYBR green PCR master mix. Reactions were run according to manufacturer protocols for at least 40 cycles. Data were analyzed using the ratiometric ΔΔCT method, and the mean relative mRNA expression for each sample was reported. All primer sequences used can be found in Additional file 1: Table S1.

### Experimental design and statistical analysis

All data are presented as the mean ± SEM. GraphPad was used for all statistical analysis. The statistical significance of the mean difference was calculated using a repeated measures ANOVA with appropriate post hoc analysis of significance. A *p* value < 0.05 was considered significant.

## Results

### Microglia exhibit regionally specific basal cytokine profiles

**Fig. 1:**
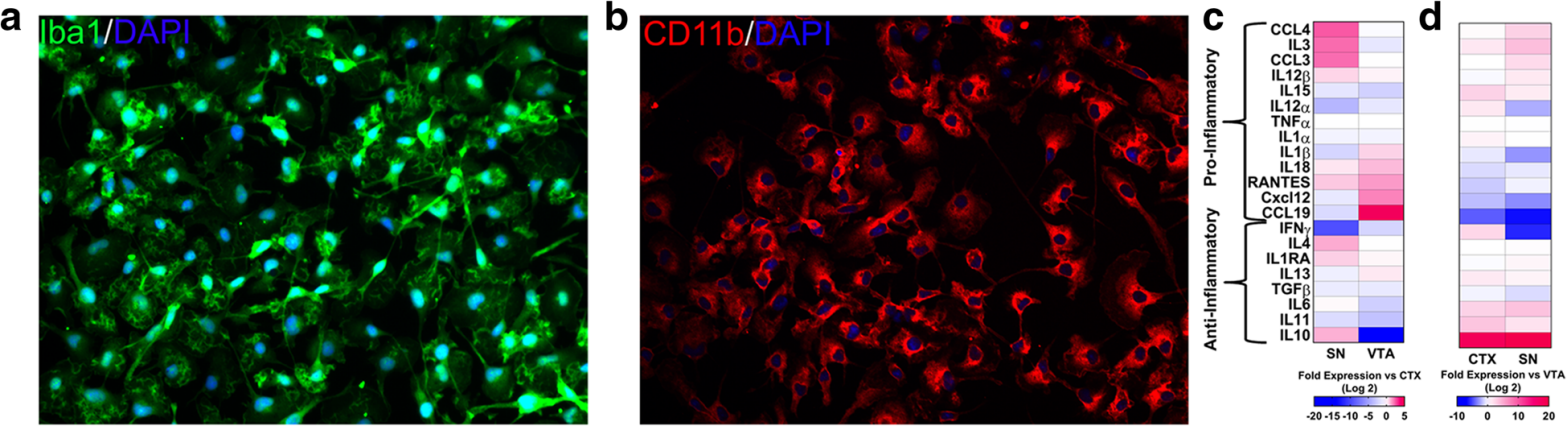
Regionally isolated microglia have distinct basal cytokine profiles. Microglia from the midbrain SN and VTA as well as the cortex (CTX) were isolated based on previously established methods. We find that cultures of microglia are pure, and the vast majority (95–100%) of cells stain for the microglial markers Iba1 (**a**) and CD11b (**b**). Regionally isolated microglia were subjected to qPCR analysis for 13 pro- and 8 anti-inflammatory cytokines and compared between different regions. In comparison to CTX microglia, SN microglia (**c**), left column, exhibit greater expression of the pro-inflammatory cytokines IL3, CCL3, and CCL4, with diminished IFNγ expression, whereas VTA microglia (c, right column) express greater levels of RANTES, Cxcl12, and CCL19, while expressing lower levels of IL10. A secondary comparison against VTA microglia (**d**) demonstrated that both SN (left column) and CTX (right column) microglia have greatly upregulated levels of IL10 and diminished expression of CCL19. A number of other differences in these profiles demonstrate a regional variability to microglia

To examine the potential regionality of microglia within the midbrain, we isolated microglia from SN and VTA and compared them to microglia from cortex (CTX) using postnatal day 1–5 transgenic rats. Microglial purity was estimated at 95–100% as the vast majority of cells stained for the microglial markers Iba1 (Fig. 1a) and CD11b (Fig. 1b). Regionally isolated microglia were then collected and examined for their basal cytokine profile. We found that regionally isolated microglia exhibit subtle differences in cytokine expression profiles (Fig. 1c, d). In comparison to isolated CTX microglia (Fig. 1c, left column), SN microglia exhibit greater levels of the pro-inflammatory cytokines IL3, CCL3, and CCL4, while exhibiting diminished expression of IFNγ. VTA microglia (Fig. 1c, right column) conversely express greater levels of pro-inflammatory RANTES, Cxcl12, and CCL19, with decreased expression of IL10. An additional comparison was performed with CTX and SN microglia against isolated VTA microglia (Fig. 1d). We found that both SN and CTX have greatly upregulated levels of IL10 and diminished expression of CCL19 (Fig. 1d). Together, these results demonstrate a diverse regionality to microglia of the midbrain based on their baseline cytokine expression levels.

### Isolated microglia have differential cytokine responses to PD mimetic toxin MPP^+^

**Fig. 2:**
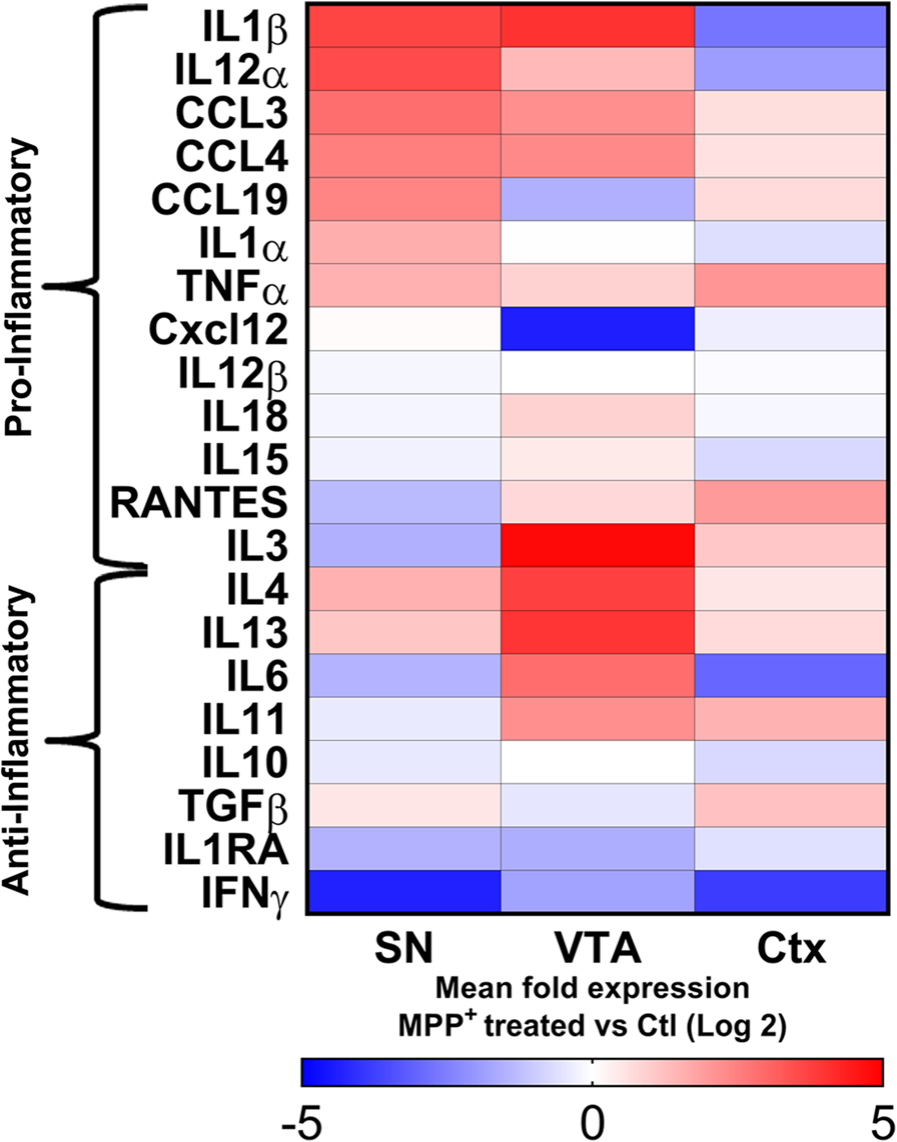
Treatment of regionally isolated microglia with the Parkinson’s toxin MPP^+^ causes differential upregulation of cytokines. Regionally isolated microglia were collected and exposed to the PD toxin MPP^+^ for 24 h. After toxin exposure, microglia were examined for expression of pro- and anti-inflammatory cytokines in comparison to untreated region-specific controls. MPP^+^-treated SN microglia (left column) exhibit a greater degree of pro-inflammatory cytokine upregulation, including IL1β, IL12α, CCL3, CCL4, CCL19, IL1α, and TNFα, coupled with significant downregulation of IFNγ compared to untreated control SN microglia. VTA microglia (middle column) share upregulation of pro-inflammatory IL1β, CCL3, CCL4, and TNFα similar to SN microglia. However, they exhibit higher levels of IL3, as well as the anti-inflammatory cytokines IL4, IL13, and IL6. Interestingly, CTX lack upregulation of IL1β and IL12α, whereas they demonstrate strong expression of TNFα

Previous studies have demonstrated that in vivo treatment with the PD toxin MPTP causes microglial activation [16, 30]. Therefore, we examined the response of our regionally isolated microglia to the active metabolite of MPTP, MPP^+^, to determine whether regionality results in differential activation profiles. We found that SN microglia (Fig. 2, left column) strongly upregulate the expression of pro-inflammatory IL1β, IL12α, CCL3, CCL4, and CCL19, while decreasing expression of anti-inflammatory IFNγ in response to toxin treatment. Interestingly, while there are some similarities in the pro-inflammatory response of VTA microglia to MPP^+^ (Fig. 2, middle column), such as upregulation of IL1β, CCL3, and CCL4, these microglia differ in their strong downregulation of Cxcl12 and significant upregulation of IL3. Finally, CTX microglia differ greatly from the other two regions examined (Fig. 2, right column). Their upregulation of pro-inflammatory cytokines is limited to TNFα and RANTES, but with strongly decreased expression of both IL1β and IL12α. However, CTX microglia mimic SN in their downregulation of anti-inflammatory IFNγ in response to MPP^+^ and also decreased expression of IL6. The differential expression of cytokines in response to MPP^+^ treatment would suggest that the addition of regionally isolated microglia would result in differential exacerbation of MPP^+^ toxicity of DA neurons.

### Regionally isolated microglia similarly exacerbate MPP^+^ toxicity

**Fig. 3:**
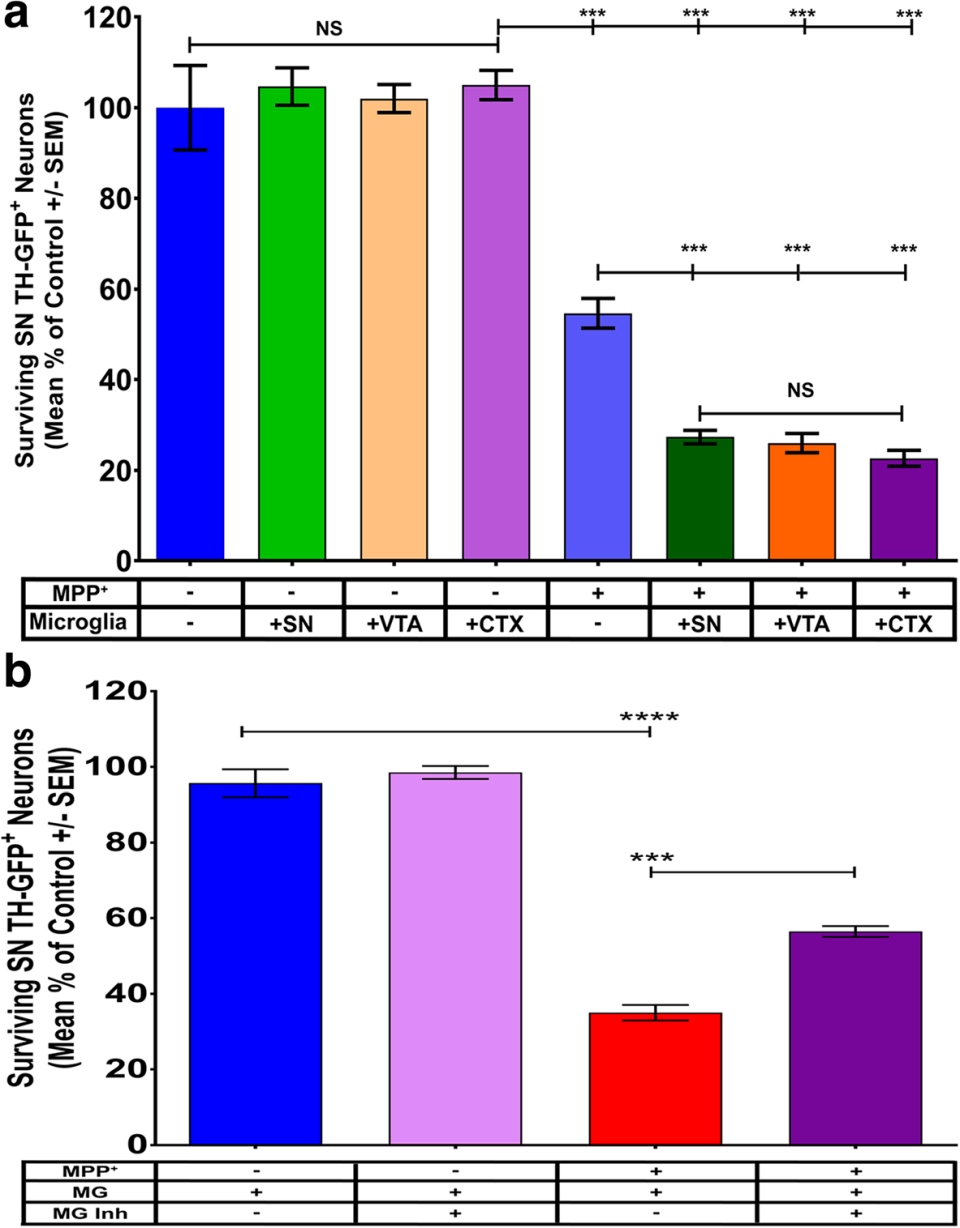
Toxicity of SN DA neurons by MPP^+^ is exacerbated in the presence of regional microglia and is alleviated by inhibition of microglial activation. **a** The addition of regionally isolated microglia (SN, VTA, CTX) to cultures of SN DA neurons and astrocytes demonstrates that the addition of microglia themselves is not deleterious to these neurons. However, coupled with MPP^+^ treatment, all regionally isolated microglia cause exacerbation of toxicity to a similar extent; one-way ANOVA with Bonferroni’s post hoc multiple comparisons test; *F* (7, 16) = 84.2, ****p < 0.0001. **b** TLR4 inhibitor C34 (light purple) does not cause any degeneration of SN DA neurons in culture. However, a significant decrease in the exacerbation of MPP^+^ toxicity is observed when microglial activation is inhibited by the drug (red compared to purple); one-way ANOVA with Tukey’s post hoc multiple comparison test; *F* (3, 14) = 144.4, ****p < 0.0001

Due to the differences seen in the cytokine profile of regionally isolated microglia, both at baseline and in response to MPP^+^, we hypothesized that regionally isolated microglia would cause differential exacerbation of MPP^+^ toxicity of midbrain DA neurons grown with their astrocytes. We find that compared to control cultures (*M* = 100, SEM = 9.3), the addition of regionally isolated microglia (SN light green, *M* = 105, SEM = 4.1; VTA tan, *M* = 102, SEM = 3.1; and CTX light purple bars, *M* = 105, SEM = 3.2) do not cause DA neuron degeneration in either SN (Fig. 3a) or VTA (data not shown, one-way ANOVA, *F* (7, 16) = 1.313 *p* = 0.3064) neuron to astrocyte cultures. Interestingly, despite the regionally associated expression differences of pro-inflammatory cytokines upon MPP^+^ stimulation, all regionally isolated microglia similarly exacerbate MPP^+^ toxicity of SN DA neurons (Fig. 3a, SN dark green, *M* = 27, SEM = 1.5; VTA orange, *M* = 26, SEM = 2.1; and CTX dark purple bars, *M* = 23, SEM = 1.8; one-way ANOVA with Tukey’s post hoc multiple comparisons test, *F* (7, 16) = 84.2, *** = *p* < 0.001), whereas VTA DA neurons (data not shown) are robustly protected from the effect of microglia in neuron to astrocyte cultures. These results suggest that VTA, but not SN, astrocytes may be protective of their regional DA neurons despite the addition of damaging microglia regardless of their region of origin.

### Inhibition of microglial activation with a TLR4 antagonist prevents exacerbation of MPP^+^ toxicity

To confirm that microglial released factors are indeed responsible for the exacerbation of MPP^+^ toxicity, we sought to prevent microglial activation by using a recently developed, commercially available TLR4 antagonist TLR4-IN-C34 (C34) [31]. When C34 is applied to cultures 2 h prior to MPP^+^ administration, we found that C34 (Fig. 3b, light purple bar, *M* = 98, SEM = 1.7) itself has no effect on the health of mixed cultures of SN neurons, astrocytes, and microglia (control, blue bar, *M* = 96, SEM = 3.7). However, coupled with MPP^+^ treatment (Fig. 3b, dark purple, *M* = 56, SEM = 1.4), we demonstrated significantly decreased exacerbation of MPP^+^ toxicity (red bar, *M* = 35, SEM = 2.1) of SN DA neurons (one-way ANOVA with Tukey’s post hoc multiple comparisons test, *F* (3, 14) = 144.4, **** = *p* < 0.0001). Cultures where C34 inhibition has occurred resemble our previously established data where microglia are absent from cultures (Kostuk et al. 2018, *in submission*). This further supports that factors released from microglia indeed exacerbate MPP^+^ toxicity of these neurons.

### Isolated VTA, but not SN, astrocytes mediate protection of both SN and VTA DA neurons from microglia

**Fig. 4:**
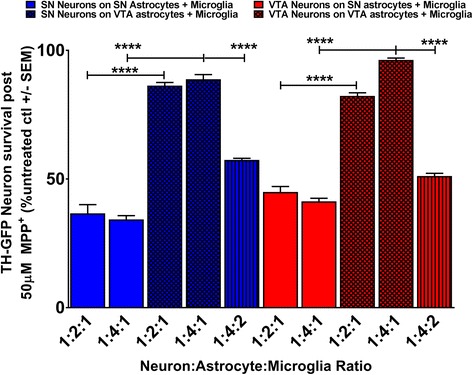
Greater ratios of isolated VTA, but not SN, astrocytes protect both SN and VTA DA neurons from MPP^+^ toxicity in the presence of microglia, but increased numbers of microglia counter this protection. Regionally homo- and heterotypic cultures were established with SN and VTA neurons/astrocytes. Greater ratios of SN astrocytes fail to increase SN (solid blue bars) or VTA (solid red bars) DA neuron survival in the presence of microglia. Conversely, VTA astrocytes robustly protect both SN (blue hatched bars) and VTA (red hatched bars) DA neurons from exacerbation of MPP^+^ toxicity when microglia are present. The protective effect of VTA astrocytes on SN (blue vertical striped bar) and VTA (red vertical striped bar) neurons is diminished when the ratio of microglia to neurons is increased from 1:1 to 2:1. Data are presented as mean ± SEM; one-way ANOVA with Tukey’s post hoc multiple comparison test; *F* (9, 28) = 270.8, ****p < 0.0001

As we demonstrated that VTA neurons are robustly protected from MPP^+^ toxicity despite the presence of microglia, we sought to determine whether this protection is due to astrocytic released factors. We created both homo- and heterotypic astrocyte-neuron cultures from the SN and VTA region in varying ratios to determine if, similar to our previous findings, greater numbers of astrocytes would afford protection despite the presence of microglia. We found that in both homotypic (SN DA neurons on SN astrocytes, Fig. 4, solid blue bars: 1:2:1 ratio *M* = 37, SEM = 3.4; 1:4:1 ratio *M* = 34, SEM 1.5) and heterotypic (VTA DA neurons on SN astrocytes, Fig. 4, solid red bars: 1:2:1 ratio *M* = 45, SEM = 2.1; 1:4:1 ratio *M* = 41, SEM = 1.2) co-cultures, higher ratios of SN astrocytes were incapable of affording greater protection of both SN and VTA neurons when microglia were present at a ratio equal to neurons. Conversely, VTA astrocytes exhibited ratio-dependent increases in protection of both SN and VTA DA neurons despite the presence of exacerbatory microglia in both homotypic (VTA DA neurons on VTA astrocytes, Fig. 4, hatched red bars: 1:2:1 ratio *M* = 82, SEM = 1.2; 1:4:1 ratio *M* = 96, SEM = 0.75) and heterotypic (SN DA neurons on VTA astrocytes, Fig. 4, hatched blue bars: 1:2:1 ratio *M* = 86, SEM = 1.2; 1:4:1 ratio *M* = 89, SEM = 1.8). To further our understanding of the protection provided by VTA astrocytes, we then sought to tip the balance towards the deleterious effects of microglia by increasing their proportion of the cells in the dish. We found that the addition of microglia at a 2:1 ratio to neurons in the presence of the most protective astrocyte ratio (4:1 astrocytes to neurons) does result in DA neuron death simply because more microglia were added (data not shown). However, when coupled with MPP^+^ treatment, we found that the protective effect of VTA astrocytes was significantly diminished (Fig. 4; SN neurons blue vertically stripped bar, *M* = 57, SEM = 0.62 and VTA neurons red vertically stripped bar, *M* = 51, SEM = 1) by increasing the ratio of microglia (one-way ANOVA with Tukey’s post hoc multiple comparisons test; *F* (9, 28) = 270.8, ****p < 0.0001). These results further suggest that microglial released factors are able to overcome the protective effect of astrocytes.

## Discussion

The regional vulnerability of SN DA neurons has been extensively studied for a number of years, with many studies focusing on the intrinsic underlying mechanisms and how these neurons differ from neighboring VTA neurons [2–5]. Recently, however, greater emphasis has been placed on understanding potential non-cell autonomous mechanisms, such as the impact of microglial cells, which may underlie this selective vulnerability.

In this study, we demonstrate that regionally isolated microglia of midbrain SN and VTA subregions differ in both their basal and MPP^+^-induced cytokine profiles. Furthermore, we demonstrate that the addition of regionally isolated microglia themselves is not deleterious to DA neurons; however, their presence significantly exacerbates MPP^+^ toxicity in SN DA neurons. Conversely, VTA DA neurons co-cultured with astrocytes are robustly protected from MPP^+^ toxicity despite the presence of microglia, suggesting that VTA astrocytes may be responsible for protection of these neurons. We further showed that pharmacological inhibition of microglial activation diminished the exacerbatory effect of microglia. Finally, we demonstrated that VTA astrocytes, but not SN, are able to protect both SN and VTA DA neurons from MPP^+^ toxicity despite the presence of deleterious microglia but that it is possible to overburden protective astrocytes with increasing numbers of microglia, thus increasing the presumptive load of microglial released factors.

The role of microglia as the immune cells of the CNS has been appreciated for a number of years [6]. Many studies have demonstrated an increased release of pro-inflammatory/neurotoxic species from microglia in response to inflammatory stimuli such as LPS [18]. Additionally, some studies have postulated a role for microglia in the progression of PD [11, 32, 33], though a debate continues as to whether microglia play a role in initiation of disease or are activated secondary to neuronal death. Supporting the latter proposition, there are a number of reports demonstrating a response of microglia to some PD toxins. Gao et al. [24] showed that the effect of rotenone, a pesticide that has been used as a PD mimetic toxin in culture, requires the presence of microglia and that this effect is dependent on the number of microglia present within the culture. Additionally, Chien et al. [34] demonstrated that microglial released cytokines and chemokines are responsible for the loss of nigrostriatal DA neurons in response to LPS. Furthermore, recent work by Smeyne et al. [16] demonstrated that systemic injection of the PD mimetic toxin MPTP results in increased microglial reactivity within the SN, suggesting that PD mimetic toxins can directly cause microglial activation and subsequent cytokine release. These findings are similar to ours in which microglial addition to DA neuron cultures exacerbates the effect of the PD mimetic toxin MPP^+^, suggesting that the release of pro-inflammatory molecules from microglia may have a vital role in the progression of PD DA neuron degeneration.

Previous studies have also demonstrated that the inhibition of microglial activation or knockdown of released cytokines from microglia may be neuroprotective to DA neurons. Pharmacological blockade of microglial activation [35–37] or knockdown of cytokines, such as IL-1 [38], demonstrates that prevention of microglial activation attenuates some of the degeneration seen in models of PD. Furthermore, the interaction of MPTP/MPP^+^ with the microglial receptor TLR4, a key receptor in the microglial activation pathway, has previously been established [39, 40]. As such, it is unsurprising that our findings demonstrate that the use of a new TLR4 inhibitor C34 [31], prior to MPP^+^ administration, results in decreased exacerbation of toxicity.

In addition to an effect of the PD mimetic toxins MPTP or MPP^+^ on the activation of microglia, a number of studies have examined the impact of alpha synuclein (α-syn) on microglia and the subsequent activation of these cells [33, 41–43]. Indeed, studies have demonstrated that α-syn is able to bind to surface receptors of microglia and result in their activation and subsequent upregulation of pro-inflammatory cytokines [44–46]. Therefore, it is plausible that the interaction of α-syn and microglia of the midbrain may be exacerbating or contributing to DA neuronal degeneration similar to that seen by the effect of MPP^+^ in our studies. However, it is still relatively unknown whether this interaction of α-syn and microglia is an initiating factor for neuronal death or secondary to the release of species from damaged or dying neurons [43]. Furthermore, a number of studies have demonstrated that microglia can act as scavengers of extracellular α-syn [47, 48], thereby clearing a potentially damaging species away from vulnerable neurons. The impact of microglial-sequestered α-syn is not fully understood, and it is possible that internalized α-syn could result in dysfunction of lysosomal pathways within microglia, similar to that seen in DA neurons [49], impairing normal functions within these cells. Therefore, the effect that α-syn may have on microglia from the SN and VTA remains an important area of investigation.

The regional variability in the transcriptional profile of microglia has been investigated previously [27]. However, potential differences of microglia within the midbrain have yet to be investigated. Our study is the first of our knowledge to sub-dissect the midbrain into the vulnerable SN and protected VTA to examine the effects of isolated microglia of these regions in a PD model. Though we demonstrate a number of basal and MPP^+^-induced cytokine differences, it is interesting to note that we fail to find any regionally specific exacerbation of MPP^+^ toxicity. A number of possible explanations exist for this fact.

First, our investigation of the transcriptional profile of these microglia was somewhat limited. We established a list of 21 canonical pro- and anti-inflammatory cytokines based on commercially available arrays. This is a very limited view of the transcriptional profile of these cells. As such, future studies utilizing more in-depth methods of investigation, such as RNAseq, are required. These methods have been used in other models, such as spinal cord injury [50], and have demonstrated a vast array of subtle injury-induced changes to microglia that should likewise be appreciated in the context of the midbrain in PD.

Secondly, the deleterious effect of these regionally isolated microglia could simply be due to a combined CCL3, CCL4, and TNFα response, as these are the three pro-inflammatory cytokines upregulated in all three regionally isolated microglial populations. However, the role of these cytokines has been investigated extensively in the context of PD [51–53], though the specific action of SN or VTA microglial-derived cytokines is still unknown. A possible course of investigation would be to selectively inhibit the production of these cytokines, or their downstream pathways, directly within these distinct midbrain nuclei and examine their effects in an in vivo PD model.

Finally, the response of microglia to inflammatory stimuli, such as LPS, is understood to be temporally dynamic. Indeed, previous studies have demonstrated that changes in the expression level of known pro-inflammatory cytokines can change even within 4 h of stimulus initiation, and these changes can be transient and disappear by 24 h after stimulus [54, 55]. Our examination of expression levels of cytokines was limited to the 24-h time point, as this is when we typically see the greatest death of DA neurons after MPP^+^ treatment. However, we cannot rule out the effect of earlier cytokine responses and their impact on DA neuronal survival. Further RNAseq studies examining the temporal gene expression changes of isolated SN, VTA, and CTX microglia are required to fully understand the role these cells may have in the initiation or exacerbation of DA neuronal death in response to MPP^+^.

The role of astrocytes as neuroprotective cells has been well established for a number of years [56–58]. Indeed, our recent work (Kostuk et al. 2018, *in submission*) has demonstrated a vast transcriptional difference between the astrocytes of the SN and VTA, suggesting that VTA astrocytes are able to mediate protection of their regional DA neurons whereas SN astrocytes lack this ability. Furthermore, the crosstalk between astrocytes, neurons, and microglia has recently become more appreciated [59–61]. Thus, the interaction of midbrain astrocytes and microglia in the context of PD pathogenesis is an important avenue of investigation. Consequently, it is unsurprising to us that greater ratios of SN astrocytes fail to mediate greater protection when deleterious microglia are present and that VTA astrocytes robustly protect both SN and VTA DA neurons from MPP^+^ toxicity despite the presence of microglia. Interestingly, however, we were able to demonstrate that it is possible to overburden protective astrocytes with greater numbers of microglia by increasing the ratio of microglia to neurons and likely their released cytokines in our cultures. This is of vital importance, as SN microglial activation and proliferation is a key feature of both human PD pathogenesis [15, 62] and animal models [16] of the disease. Therefore, as the concentration of microglial factors increases during the course of disease, less protective astrocytes in the SN may become more overtaxed by significant increases of microglial-released cytokines. In contrast, VTA astrocytes may be better suited to balance against these deleterious factors as there are fewer microglia and greater proportions of protective astrocytes present basally [25, 26, 63, 64], and thus, microglial activation or proliferation may never reach the critical threshold needed to cause VTA DA degeneration.

## Conclusions

In summary, the data in this paper suggests that regional differences in vulnerability of midbrain DA neurons to the PD toxin MPP^+^ may rely on a delicate balance between protective factors supplied by astrocytes and deleterious cytokines released by microglia. We demonstrate that regionally isolated microglia of the SN, VTA, and CTX differ in their basal and MPP^+^-induced cytokine profiles. Despite these transcriptional differences, all regionally isolated microglia exacerbate MPP+ toxicity of SN DA neurons, and this exacerbation can be attenuated by inhibition of microglia activation. Furthermore, VTA, but not SN, astrocytes are able to mediate protection from microglial-released factors, though it is possible to overcome the protective function of astrocytes with greater numbers of microglia. Thus, these findings help to expand our current view of this neurodegenerative disease process and highlight the need for greater understanding of the balanced role of astrocytes and microglia in the midbrain.

## Additional file

Additional file 1: **Table S1.** List of primers used for qRT-PCR. (DOCX 29 kb)

## Abbreviations

α-syn: Alpha synuclein
CTX: Cortex
CCL: Chemokine (C-C motif) ligand
DA: Dopaminergic
IL: Interleukin
IFNγ: Interferon gamma
LPS: Lipopolysaccharide
MPTP: 1-Methyl-4-phenyl-1,2,3,6-tetrahydropyridine
MPP^+^: 1-Methyl-4-phenylpyridinium
PD: Parkinson’s disease
SN: Substantia nigra
TNFα: Tumor necrosis factor alpha
TLR4: Toll-like receptor 4
VTA: Ventral tegmental area
