# Supplementary material for: Regional microglia are transcriptionally distinct but similarly exacerbate neurodegeneration in a culture model of Parkinson’s disease
Source: J Neuroinflammation. 2018 May 11;15:139. doi: 10.1186/s12974-018-1181-x (PMC5948771; doi:10.1186/s12974-018-1181-x)
Supplement: Supplementary file 1 — Table S1. List of primers used for qRT-PCR. (DOCX 29 kb) [file 12974_2018_1181_MOESM1_ESM.docx]

Supplemental Table 1 – Rat qPCR primer sequences

| Gene | Sequence |
| --- | --- |
| GAPDH Fwd | CTCAGTTGCTGAGGAGTCCC |
| GAPDH Rev | ATTCGAGAGAAGGGAGGGCT |
| IL1a Fwd | GTGGTGTCAGCAACATCAAACA |
| IL1a Rev | TTCTGGAAGCTGTGAGGTGC |
| IL1b Fwd | TGGAGCTTCCAGGATGAGGACC |
| IL1b Rev | GTTCATCTCGAAGCCTGCAGTG |
| IL3 Fwd | TTCAGACAGGGGCTCAGATG |
| IL3 Rev | GTCCAGGTTTACTCTCCGCA |
| IL4 Fwd | ATCATCAACACTTTGAACCAGGTC |
| IL4 Rev | ACCCTGGAAGCCCGACAGATGA |
| IL6 Fwd | TACCACTTCACAAGTCGGAGG |
| IL6 Rev | CTGACAGTGCATCATCGCTGTTC |
| IL10 Fwd | CGGGGTGACAATAACTGCACCC |
| IL10 Rev | CTGTCAGCAGTATGTTGTCCAGC |
| IL11 Fwd | GCTTCCTGGAGTGCTGACAA |
| IL11 Rev | GTAAGCGACGAAGTAGCCGT |
| IL12a Fwd | ATGAGAGTTGCCTGGCTACTAA |
| IL12a Rev | CCTCATAGATGCTACCAAGGCAC |
| IL12b Fwd | AGACCCTGCCCATTGAACTG |
| IL12b Rev | CAGGAGTCAGGGTACTCCCA |
| IL13 Fwd | AACAGCAGCATGGTATGGAGCG |
| IL13 Rev | TGGGTCCTGTGGATGGCATTGC |
| IL15 Fwd | GTAGGTCTCCCTAAAACAGAGGC |
| IL15 Rev | TCCAGGAGAAAGCAGTTCATTGC |
| IL18 Fwd | ACCCGCCTGTGTTCGAGGACATG |
| IL18 Rev | TGTTTTTACAGGAGAGGGTAGAC |
| CCL3 Fwd | ACTTCCTGCTGCTTCTCTTACA |
| CCL3 Rev | ACCACAGCTGGCTGGGAGCAAA |
| CCL4 Fwd | ACCCTCCCACTTCCTGCTGCTT |
| CCL4 Rev | TTGCCTGCCTTTTTTGGTCAGA |
| CCL19 Fwd | GGTGCTAACGATGCGGAAGA |
| CCL19 Rev | ACAGAGCTGGTAGCCCCTTA |
| Cxcl12 Fwd | CATCCAGAGCTTGACGGTGACC |
| Cxcl12 Rev | GGCTTCAGGGTTGAGACAAACT |
| RANTES Fwd | CCTGCTGCTTTGCCTACCTCTC |
| RANTES Rev | ACACACTTGGCGGTTCCTTCGA |
| IL1RA Fwd | TGTGCCTGTCTTGTGTCAAGTC |
| IL1RA Rev | GCCTGTCTCGGAGCGGATGAAG |
| TNFa Fwd | GGTGTCTGTGCCTCAGCCTCTT |
| TNFa Rev | GCCATGGAACTGATGAGAGGGAG |
| TGFb Fwd | TGATACGCCTGAGTGGCTGTCT |
| TGFb Rev | CACAAGAGCAGTGAGCACTGAA |
| IFNg Fwd | CAGCAACAGTAAAGCAAAAAAGG |
| IFNg Rev | TTTCCGCTTCCTTAGGCTAGAT |
